# Comparative effectiveness of acupuncture, fire needle, and herbal therapies for melasma: A Bayesian network meta-analysis

**DOI:** 10.1097/MD.0000000000048240

**Published:** 2026-04-17

**Authors:** Yangyingqi Dai, Guangye Li, Pingsheng Hao

**Affiliations:** a Department of Dermatology, Hospital of Chengdu University of Traditional Chinese Medicine, Chengdu, Sichuan Province, China; b Department of Orthopaedics, Hospital of Chengdu University of Traditional Chinese Medicine, Chengdu, Sichuan Province, China.

**Keywords:** acupuncture, Bayesian network meta-analysis, external therapy, fire needle, melasma, TCM

## Abstract

**Background:**

Melasma is a prevalent pigmentary disorder among reproductive-age Asian women. Although various treatment modalities exist, most demonstrate limited efficacy and carry the risk of adverse effects. In recent years, external Traditional Chinese Medicine (TCM) therapies (including acupuncture, fire needle, and herbal external applications) have attracted increasing attention as alternative interventions. However, their comparative effectiveness remains uncertain due to the lack of systematic evaluation.

**Methods:**

A Bayesian network meta-analysis was conducted to assess the relative efficacy of different external TCM therapies for melasma. A total of 10 randomized controlled trials involving 944 participants were included. Primary outcomes were changes in melasma lesion area and pigmentation color. Subgroup analyses and surface under the cumulative ranking probabilities were employed to compare intervention rankings. Heterogeneity, publication bias, and consistency were assessed using standard meta-analytic methods.

**Results:**

Compared with conventional treatments, external TCM therapies (including body acupuncture, fire needle, and herbal applications) did not demonstrate statistically significant improvements in melasma area or pigmentation color (all mean differences included or approached zero). Subgroup analysis indicated that fire needle therapy may offer relative improvement in pigmentation, while body acupuncture combined with conventional treatment showed a slight advantage in reducing lesion area; however, the overall strength of evidence was low. The presence of study heterogeneity, small sample sizes, and potential publication bias likely contributed to the lack of statistical significance.

**Conclusion:**

Current evidence does not support a definitive advantage of external TCM therapies over conventional treatments in the management of melasma. Clinical decision-making should be individualized, and the application of external TCM therapies should be considered with caution. High-quality, large-sample, and methodologically standardized randomized controlled trials are warranted to further explore the therapeutic potential of these interventions.

## 1. Introduction

Melasma is a chronic acquired pigmentary skin condition prevalent among reproductive-age Asian women.^[1]^ Although past research has identified genetic factors, ultraviolet radiation, and sex hormones as primary causes, recent studies also implicate blue light, air pollution, and psychological stress as contributing factors.^[2]^

Current understanding of melasma pathogenesis involves melanocytes, fibroblasts, and sebaceous gland cells, with increased pigment synthesis and impaired transport, basement membrane disruption, vascular proliferation and blood stasis, inflammatory responses, compromised skin barrier, and photoaging.^[3,4]^ Current melasma management strategies include avoiding triggers and sun protection, systemic medications like tranexamic acid, vitamins C and E, glutathione, and topical treatments such as hydroquinone, retinoids, azelaic acid, and tranexamic acid. Stable melasma often involves combined treatments with chemical peels, phototherapy, and lasers.^[5]^

While chemical peels, laser treatments, phototherapy, and dermabrasion may offer some improvement, their evidence of effectiveness remains limited, and they frequently cause post-inflammatory hyperpigmentation, particularly in darker-skinned individuals. Medication remains the mainstay of melasma therapy. The triple combination of 4% hydroquinone, 0.05% tretinoin, and 0.01% fluocinolone acetonide remains the only Food and Drug Administration-approved gold-standard treatment.^[6]^ Oral tranexamic acid, alone or combined, can also reduce melasma area and severity index scores. Additionally, various cosmetics and plant extracts for skin brightening are beneficial. Physical sunscreens containing zinc oxide, iron oxide, titanium dioxide, and silicone offer photoprotection and camouflage effects. Thus, combined therapies are generally considered the most effective.

External Traditional Chinese Medicine (TCM) treatments for chloasma, including acupuncture, moxibustion, and external application of Chinese medicine, are increasingly attracting the attention of clinical researchers.^[7]^ Many clinical trials have been published on the effectiveness of external TCM treatments for chloasma, but the differences in their efficacy are still unclear.^[8]^ At the same time, we also noticed that there are unfinished randomized controlled trials (RCTs) of acupuncture treatment for chloasma registered on the clinical trial registration platform (clinicaltrials.gov), indicating that the effectiveness of external TCM treatments for chloasma is still a research hotspot. Therefore, this study is based on published RCTs and uses Bayesian network meta-analysis to study the effectiveness of different external TCM treatments, try to find the best external TCM treatment or treatment plan, and provide more reference value for clinical application and research.

## 2. Materials and methods

### 2.1. Study registration

This study was conducted according to the reporting guidelines for systematic reviews and net meta-analysis (PRISMA-NMA) and was prospectively registered with PROSPERO (ID: CRD42024503938).

### 2.2. Literature search strategy

We systematically searched for RCTs of external Chinese medicine treatments for melasma published in PubMed, Sinomed, Embase, Cochrane, Wipro, Wanfang, and China Knowledge Networks, with the search ending on September 10, 2024, using a combination of subject terms and free terms. Search terms included Melanosis, Acupuncture, Acupuncture Therapy, Acupuncture, Ear, Moxibustion, and so on.

### 2.3. Inclusion and exclusion criteria

#### 2.3.1. Inclusion criteria

Population: Patients definitively diagnosed with melasma, without restrictions on ethnicity, nationality, gender, age, or disease duration.

Intervention: External TCM therapies (acupuncture, moxibustion, scraping, herbal external applications).

Comparison: Conventional treatment.

Outcome: Melasma area and color.

Study design: RCTs.

#### 2.3.2. Exclusion criteria

Studies with significantly flawed diagnoses, irrelevant interventions, lacking appropriate comparison or outcome measures, and non-RCTs.

### 2.4. Data extraction

To ensure the accuracy and rigor of data extraction, 2 independent reviewers extracted relevant data based on predefined criteria. Extracted information included study authors and publication year, baseline characteristics, intervention details, and outcome measures, all of which were recorded in an Excel spreadsheet. After initial extraction, the data were cross-checked and consolidated by the 2 reviewers.

Next, all retrieved references were imported into Zotero for deduplication. The remaining records were screened by title and abstract, and studies that did not meet the inclusion criteria (such as review articles and conference abstracts) were excluded.

Finally, the full texts of potentially eligible articles were reviewed in detail. Studies were excluded if they had uncleared diagnostic criteria, incomplete outcome data, or duplicate publications. In cases of disagreement, a third reviewer with extensive experience in evidence-based medicine was consulted to reach consensus.

### 2.5. Risk of bias assessment

**Figure 1.**
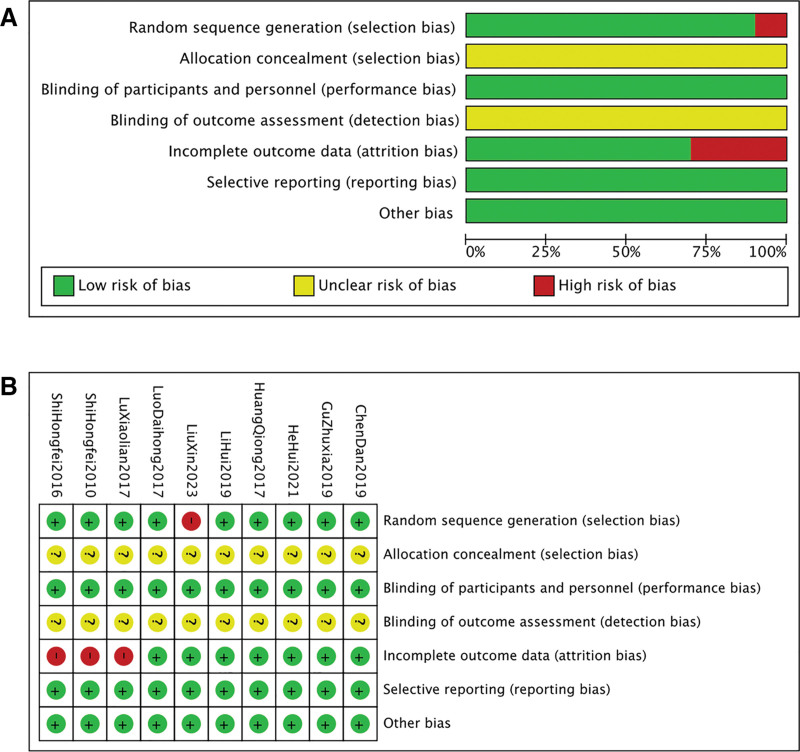
The risk of bias assessment. (A) Risk of bias graph; (B) Risk of bias summary.

Two reviewers (Yangyingqi Dai& Guangye Li) independently assessed the risk of bias using the Cochrane Risk of Bias tool for RCTs. The assessment covered the following 7 domains: generation of the random sequence, allocation concealment, blinding of participants and personnel, blinding of outcome assessors, completeness of outcome data, selective reporting, and other potential sources of bias. Each item was rated as having a low, high, or unclear risk of bias. The results were visually presented using Revman Version 5.4 (The Cochrane Collaboration, The Nordic Cochrane Centre, Copenhagen, Denmark). (Fig. 1)

A comprehensive risk of bias assessment across all included studies revealed the following methodological characteristics. Regarding random sequence generation (selection bias), most studies implemented standardized subgroup randomization procedures, though 1 study exhibited potential flaws in this domain. Allocation concealment (selection bias) remained indeterminate across all studies due to unreported methodological details (e.g., sealed envelopes, centralized randomization systems), resulting in an unclear risk of selection bias. All studies demonstrated low-risk of performance bias through adequate blinding of participants and personnel. Conversely, blinding of outcome assessment (detection bias) could not be evaluated as no study documented methodological specifics, warranting an “unclear risk” designation. For incomplete outcome data (attrition bias), 3 studies presented high risk due to substantial missing data and inadequate handling, while others maintained low-risk given minimal missing data and appropriate analytical approaches. Selective reporting (reporting bias) was uniformly low-risk, as all studies comprehensively reported prespecified endpoints and analytical outcomes without evidence of results screening. No additional sources of bias were identified. Collectively, most studies exhibited robust methodology in randomization, performance bias control, and reporting practices, supporting the reliability of their core findings. However, results from studies with randomization deficiencies (n = 1) and significant attrition issues (n = 3) require cautious interpretation. Furthermore, inherent challenges in blinding and allocation concealment for manual therapies (e.g., acupuncture, scraping) rendered all studies “unclear risk” in these domains, which may further weaken the overall evidence strength.

### 2.6. Outcome measures

The primary outcome measures were melasma color and affected area.

### 2.7. Statistical analysis

#### 2.7.1. Software

Risk of bias was assessed using Revman 5.2 (Copenhagen, Denmark), provided by the Cochrane Collaboration. A network geometry plot was generated using Stata 14.2 (StataCorp LLC, College Station), and comparison-adjusted funnel plots for publication bias were produced in RStudio (version 2022.02.0; Posit PBC, Boston).

#### 2.7.2. Network meta-analysis

A Bayesian random-effects model was applied to compare the relative effectiveness of different interventions. Markov Chain Monte Carlo methods were used for modeling, with 4 chains running simultaneously. The burn-in period was set at 20,000 iterations, followed by 50,000 iterations for simulation. The Deviance Information Criterion was used to assess model fit and global consistency. When closed loops existed in the network, node-splitting methods were employed to evaluate local inconsistency.

All interventions were ranked using the surface under the cumulative ranking probabilities, and league tables were generated to compare effect sizes between interventions.

Given that several included randomized controlled trials (RCTs) conducted multi-arm studies using the same TCM intervention with varying treatment durations and frequencies, we conducted a Bayesian network meta-regression to examine whether these factors significantly influenced efficacy compared to conventional treatments. When the number of studies for a specific outcome was ≥ 10, comparison-adjusted funnel plots were used to visually assess potential publication bias. All statistical analyses were performed using R (version 4.4.0; The R Foundation for Statistical Computing, Vienna, Austria), with a *P* value < .05 considered statistically significant.

## 3. Results

### 3.1. Literature search and study selection process

A total of 2230 articles were initially identified through searches in both Chinese and English databases. After removing 720 duplicate articles, 1510 remained. Among these, 278 articles unrelated to chloasma and external Chinese medicine treatments, 413 non-RCT studies, and 109 dissertations were excluded. Additionally, 1 article was not retrieved. Eventually, 710 articles underwent full-text review, with 189 reviews, 20 animal experiments, 475 case reports, 13 meta-analyses, and 2 guidelines excluded. Finally, 10 RCTs were included in this meta-analysis.

### 3.2. Quality assessment of included studies

Regarding randomization methods, 4 studies reported using random number tables, while 4 studies did not. None of the included studies mentioned the use of allocation concealment. Blinding was implemented in 9 of the studies. All studies reported complete outcome data, and no evidence of selective reporting was identified.

### 3.3. Results of the network meta-analysis

**Figure 2.**
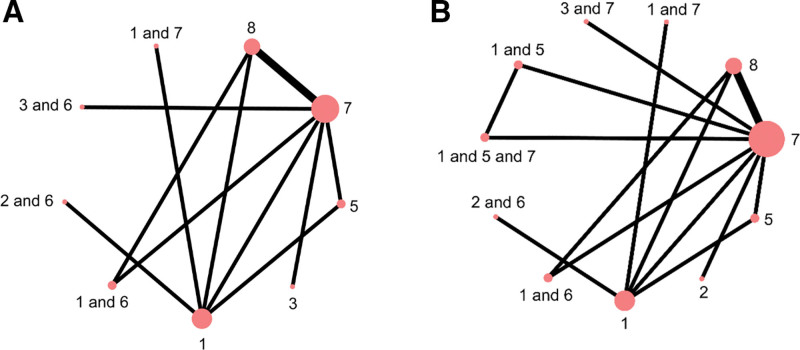
Results of the network meta-analysis. Network of interventions for melasma (A) Outcome: Lesion area; (B) Outcome: Color score.

A total of 10 studies reported on 11 different interventions. Figure 2 illustrates the network evidence diagram for each outcome. The size of each node represents the total number of participants receiving that intervention, and the thickness of the connecting lines indicates the number of direct comparisons between interventions. Intervention 7 included the highest number of participants, and the most frequent direct comparisons occurred between interventions 7 and 8. Multiple closed loops were formed within the network, indicating that most studies were closely interconnected.

#### 3.3.1. Melasma area

##### 3.3.1.1. Connections between interventions

**Figure 3.**
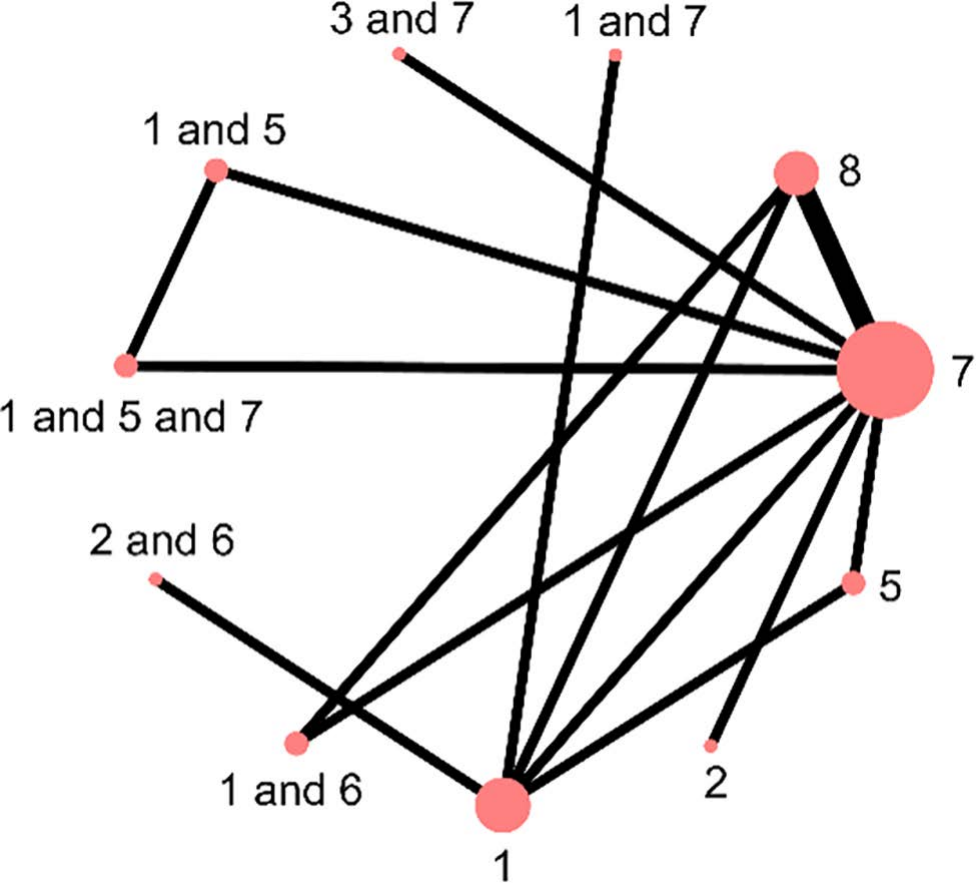
Associations between interventions.

Nine studies reported outcomes related to melasma-affected area, covering 5 types of external TCM interventions and 1 placebo. These interventions included body acupuncture, facial acupuncture, body acupuncture combined with auricular acupoint therapy, facial acupuncture combined with auricular therapy, herbal external application, body acupuncture combined with herbal medicine, body acupuncture and herbal therapy in combination with conventional treatment, and fire needle therapy combined with conventional treatment. Among them, body acupuncture, herbal applications, and auricular therapy formed closed loops, with tight interconnections across the included studies. (Fig. 3)

##### 3.3.1.2. Synthesized results

**Figure 4.**
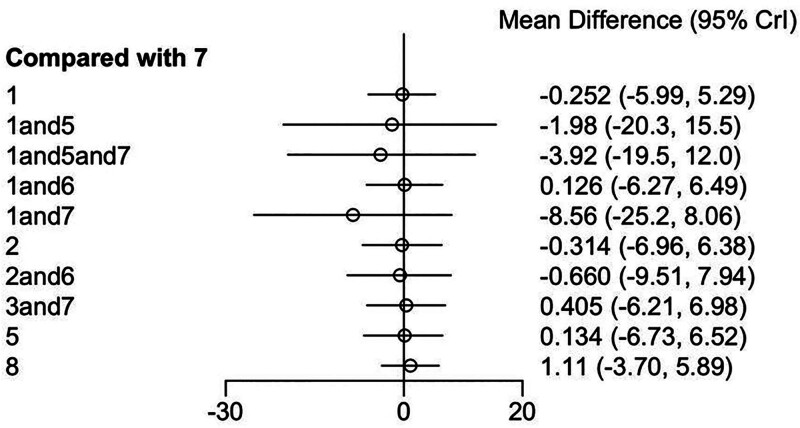
Synthesized results forest plot. Crl = credible interval.

Overall, from this forest plot, the mean differences between all comparison groups and the reference group (conventional treatment group) were not statistically significant. (Fig. 4)

#### 3.3.2. Color

**Figure 5.**
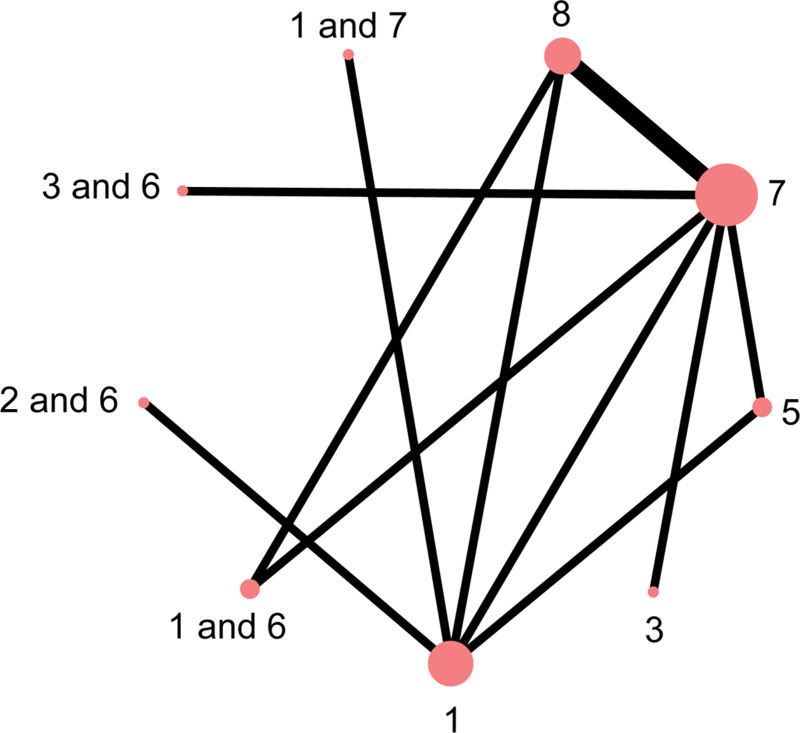
Melasma color treatment.

Eight studies reported outcomes related to melasma color improvement, involving 5 types of external TCM therapies and 1 placebo. The interventions included fire needle, body acupuncture, combinations of body acupuncture, facial acupuncture, and fire needle with auricular point therapy, as well as herbal external application, body acupuncture alone, and body acupuncture combined with herbal medicine. Among these, body acupuncture, herbal therapy, and auricular therapy formed closed loops, with strong interconnections across studies. (Fig. 5)

**Figure 6.**
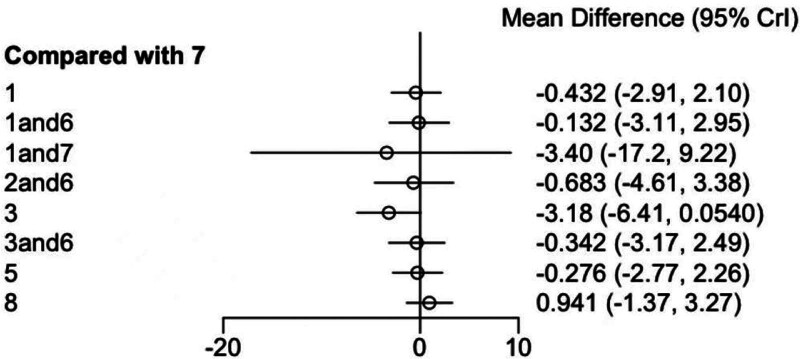
Forest plot of comparisons vs intervention. Crl = credible interval.

One comparison yielded an effect size of −3.18 with a 95% confidence interval (CI) of (−6.41, 0.0540). Although the CI approaches inclusion of zero, it does not fully include it, suggesting the mean difference between this intervention and the reference group may be of borderline statistical significance, and the trend appears to favor a potential effect. (Fig. 6)

Overall, as shown in the forest plot, most comparison groups (7 out of 10) showed no statistically significant differences in mean change compared to the control group. Only 3 comparisons exhibited results that were close to statistical significance, yet these findings should be interpreted with caution.

## 4. Discussion

**Figure 7.**
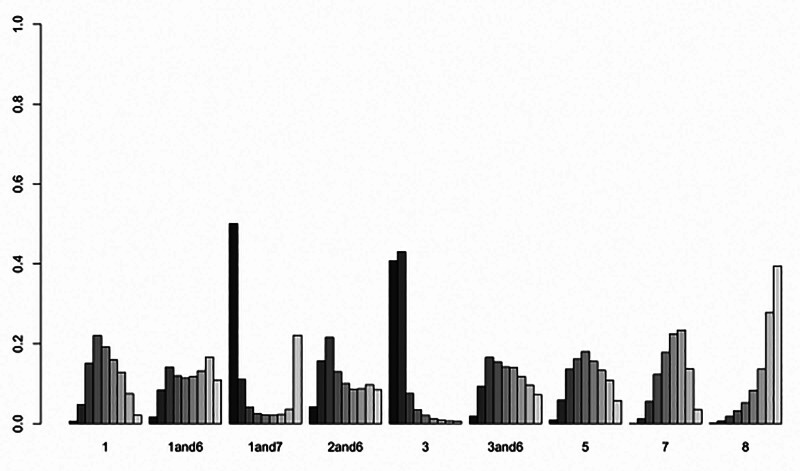
Cumulative ranking curve.

This meta-analysis included 10 relevant studies and aimed to evaluate the effects of external TCM therapies (including body acupuncture, facial acupuncture, fire needle, scraping therapy, herbal application, and auricular therapy) on melasma color and lesion area. However, the overall analysis revealed no statistically significant differences between these 6 external TCM modalities and conventional treatments in improving melasma color or area. Notably, this finding contrasts with several previously published studies, which reported significant benefits of acupuncture and other TCM interventions in improving melasma symptoms. (Fig. 7)

Although no statistically significant differences were found among the treatment groups in terms of color improvement, surface under the cumulative rankings suggested that fire needle therapy may have a relative advantage in enhancing pigmentation appearance. In addition, body acupuncture combined with conventional therapy appeared more effective in reducing lesion area.

Acupuncture is an external therapy rooted in the principles of regulating internal organ function and restoring systemic balance. Through the stimulation of specific acupoints, acupuncture promotes meridian flow and harmonizes the circulation of qi and blood, thereby nourishing the facial skin and alleviating pigmentation symptoms. Furthermore, acupuncture is believed to modulate the functional status of the liver, spleen, and kidneys, which may help restore systemic homeostasis and contribute to the resolution of melasma.

Beyond lightening pigmentation, acupuncture can enhance skin metabolism, brighten overall skin tone, and improve texture. It also regulates the endocrine system by balancing the metabolism of estrogen and progesterone, thereby addressing the underlying hormonal disturbances associated with melasma. Additionally, acupuncture may relieve comorbid conditions such as menstrual irregularities, constipation, insomnia, and mood disturbances: all of which are commonly linked to endocrine imbalance; thus improving patients’ overall quality of life.

When acupuncture is combined with conventional treatment, its ability to regulate endocrine and organ function may help normalize hormonal levels, while conventional therapies concurrently suppress melanin synthesis and promote melanin degradation. This synergistic approach often leads to more noticeable improvements in both the color and area of melasma, enhancing treatment efficacy. Moreover, acupuncture-induced improvements in local blood circulation may facilitate deeper skin penetration of topical agents, thereby maximizing their therapeutic effects. For example, when hydroquinone cream is used, improved circulation can enhance its delivery to melanocytes, suppressing their activity and potentiating the overall treatment outcome.

Fire needle therapy is a specialized acupuncture technique in which a heated needle is rapidly inserted into the skin. This technique stimulates local vasodilation, improving blood flow in affected regions. Enhanced circulation promotes better oxygen and nutrient delivery to the skin, accelerating cellular metabolism and facilitating the clearance of metabolic waste and pigment deposits. For instance, improved blood flow may help melanocyte-derived melanin granules to be more effectively broken down and metabolized, thereby reducing pigmentation.^[9,10]^

Previous meta-analyses and network meta-analyses have suggested that acupuncture and scraping therapy may outperform conventional treatments. For example, a meta-analysis by Yao Songling et al on scraping therapy for melasma found significant improvements in total clinical effectiveness, lesion area, and pigmentation scores (relative risk = 1.23, 95% CI [1.16, 1.30], Z‑statistic = 7.14, *P* < .00001), with outcomes superior to the control group.^[11]^ Similarly, a study by Wu Guiling et al showed that acupuncture therapy had greater total efficacy and better performance in improving skin lesion scores than pharmacologic treatments (relative risk = 1.32, 95% CI [1.26, 1.39], *P* < .01).^[12]^

The lack of statistically significant findings in our meta-analysis may be attributable to several key factors.

### 4.1. Study heterogeneity

#### 4.1.1. Differences in study design

Although all studies included in this meta-analysis were RCTs, the quality of randomization procedures varied, and sample sizes were generally small. These limitations may introduce biases like those observed in non-randomized studies and contribute to heterogeneity, potentially obscuring the true effects of the interventions.

#### 4.1.2. Variability in participant characteristics

The included studies enrolled participants with a wide age range and varying degrees of melasma severity. Due to the lack of standardized grading criteria for melasma, disease severity was inconsistently reported across studies. Patients with mild symptoms may have responded better to external TCM therapies, whereas those with more severe cases may have shown limited responsiveness, possibly diluted the observed effect sizes and resulted in an overall negative outcome.

#### 4.1.3. Differences in intervention implementation

Substantial variation was observed in the execution of external TCM therapies across studies. For example, in body acupuncture treatments, the selection of acupoints often differed depending on syndrome differentiation, and treatment duration and frequency were not standardized. Similarly, in studies involving herbal external applications, the types and dosages of herbal formulations varied. Even the conventional treatments used as comparators (such as topical hydroquinone or laser therapy) were inconsistent across trials. These methodological differences likely led to inconsistent therapeutic effects and may have limited the ability of the meta-analysis to detect significant overall benefits.

### 4.2. Sample size limitations

Although this meta-analysis included 10 studies involving a total of 944 patients, the overall sample size remains relatively modest. A limited sample size reduces statistical power and may hinder the detection of subtle but clinically relevant differences between intervention and control groups. This constraint may have prevented the identification of modest benefits of external TCM therapies on melasma color and area, thereby contributing to the negative results.

In addition, some of the included studies had small individual sample sizes and were therefore more susceptible to random variation. When such small sample studies are combined with larger studies in meta-analyses, their inherent variability may weaken the overall signal, further contributing to the null findings.

### 4.3. Publication bias

This meta-analysis could not definitively rule out the potential for publication bias. The risk of bias assessment indicated that, beyond concerns related to randomization, performance bias (blinding), and reporting bias (where most studies demonstrated low-risk), several studies exhibited high risk attributable to methodological deficiencies in random sequence generation and significant attrition bias from problematic handling of missing data. Furthermore, inherent operational constraints of manual therapies (e.g., acupuncture, scraping) rendered allocation concealment and blinding of outcome assessment universally “unclear risk” across all included studies, likely compromising the overall evidence strength. Crucially, the potential non-publication or delayed publication of studies reporting null findings regarding the efficacy of external TCM therapies for melasma may have resulted in an incomplete evidence base. Consequently, the available published literature might not fully represent the true clinical landscape, potentially leading to an overestimation of the pooled treatment effect in this meta-analysis.

### 4.4. Clinical implications

The negative findings of this meta-analysis suggest that, based on the current body of evidence, there is insufficient support to confirm a significant advantage of external TCM therapies over conventional treatments in improving melasma color and lesion area. Therefore, clinicians should make individualized treatment decisions, considering patient-specific factors, available therapeutic options, and the need for further high-quality research.

Consistent with some previous studies, this analysis also failed to demonstrate a statistically significant benefit of external TCM interventions in melasma treatment, thereby reinforcing the notion that such therapies may not consistently yield superior outcomes in this context. However, a few earlier studies have reported positive results. These discrepancies may be attributed to the heterogeneity, limited sample sizes, and other methodological limitations, and merit further investigation.

### 4.5. Implications for future research

#### 4.5.1. Improvement in study design

Future research should aim to adopt more standardized and rigorous randomized controlled trial designs. Ensuring robust randomization procedures and adequate sample sizes will help reduce methodological heterogeneity, enhance statistical power, and allow for more accurate detection of the potential therapeutic effects of external TCM interventions.

#### 4.5.2. Sample size planning

To avoid false-negative outcomes due to insufficient sample size, future studies should pre-calculate appropriate sample sizes based on expected effect sizes and desired statistical power. This will ensure that even modest differences between TCM and control interventions can be detected with sufficient precision.

#### 4.5.3. Control of publication bias

To better reflect real-world effectiveness, future research should include prospective trial registration and promote the publication of negative or null results. This approach will help reduce publication bias and improve the comprehensiveness and objectivity of evidence synthesis.

## 5. Conclusion

In summary, the negative findings of this meta-analysis are likely due to a combination of methodological heterogeneity, limited sample size, and potential publication bias. These results provide important insights for clinical practice and highlight key areas for methodological improvement in future research. It is anticipated that upcoming high-quality studies will offer a more accurate evaluation of the therapeutic potential of external TCM interventions for melasma.

## Acknowledgments

We would like to express our sincere gratitude to all individuals and organizations who supported and assisted us throughout this meta-analysis. Special thanks to the following authors: Pinghseng Hao. Without them, this research would not have been possible.

## Author contributions

**Conceptualization:** Yangyingqi Dai.

**Data curation:** Yangyingqi Dai, Guangye Li.

**Formal analysis:** Yangyingqi Dai.

**Investigation:** Yangyingqi Dai.

**Methodology:** Yangyingqi Dai.

**Project administration:** Yangyingqi Dai.

**Resources:** Yangyingqi Dai.

**Software:** Yangyingqi Dai, Guangye Li.

**Supervision:** Yangyingqi Dai, Pingsheng Hao.

**Validation:** Yangyingqi Dai.

**Visualization:** Yangyingqi Dai.

**Writing – original draft:** Yangyingqi Dai, Guangye Li.

**Writing – review & editing:** Yangyingqi Dai, Guangye Li, Pingsheng Hao.
